# A study of an adaptive replication framework for orchestrated composite web services

**DOI:** 10.1186/2193-1801-2-511

**Published:** 2013-10-05

**Authors:** Marwa F Mohamed, Hany F ElYamany, Hamed M Nassar

**Affiliations:** Computer Science Department, Faculty of Computers and Informatics, Suez Canal University, 41522 Ismailia, Egypt; Electrical and Computer Engineering Department, Beirut Arab University, Beirut, Lebanon

**Keywords:** Service-Oriented Architecture (SOA), Replication, Composite web service, Orchestration, QoS, Load balancing

## Abstract

Replication is considered one of the most important techniques to improve the Quality of Services (QoS) of published Web Services. It has achieved impressive success in managing resource sharing and usage in order to moderate the energy consumed in IT environments. For a robust and successful replication process, attention should be paid to suitable time as well as the constraints and capabilities in which the process runs. The replication process is time-consuming since outsourcing some new replicas into other hosts is lengthy. Furthermore, nowadays, most of the business processes that might be implemented over the Web are composed of multiple Web services working together in two main styles: Orchestration and Choreography. Accomplishing a replication over such business processes is another challenge due to the complexity and flexibility involved. In this paper, we present an adaptive replication framework for regular and orchestrated composite Web services. The suggested framework includes a number of components for detecting unexpected and unhappy events that might occur when consuming the original published web services including failure or overloading. It also includes a specific replication controller to manage the replication process and select the best host that would encapsulate a new replica. In addition, it includes a component for predicting the incoming load in order to decrease the time needed for outsourcing new replicas, enhancing the performance greatly. A simulation environment has been created to measure the performance of the suggested framework. The results indicate that adaptive replication with prediction scenario is the best option for enhancing the performance of the replication process in an online business environment.

## Introduction

Software architecture is a set of structures that aims to understand the software capabilities and efficiency. Basically, it defines and structures a software system into interrelated and well-established components (Len et al. 2003; May et al. 2009). Service-Oriented Architecture (SOA) is a particular architectural style to decompose the software system into a set of reusable, scalable, interoperable and business-encapsulated components, typically called Web services (Thomas 2005). Web services (WS) are published, described, discovered and accessed using several standard protocols including WSDL for service description, SOAP for intercommunication, HTTP for binding data over networks, and UDDI for service registration and discovery (Papazoglou 2007).

In the last decade, the number of web services has increased steadily and the Business-to-Business (B2B) (Albert Napier et al. 2005) applications that demand them have proportionally increased. Nowadays, the global market advocates web services vendors to integrate and work together under specific rules and constraints to implement complex business transactions that fulfill all customers’ requirements. This type of web service integration and combination is called web service composition. In particular, there are a couple ways to composite web services: orchestration and choreography (Abdaldhem et al. 2009). In orchestration, a central component, called orchestrator, controls the communication and interaction among the different running web services in an SOA environment. The invoked web services may not know that it is involved in a composite process. Choreography, by contrast, has no central component, and the web services certainly recognize that they are involved in a composition process and therefore, can exchange messages directly among themselves.

WS providers are obligated to enhance the performance and availability of the exposed web service in the market to quickly and effectively respond to all incoming requests. Due to the huge number of messages that a WS may receive in a short time, the WS can fail or become intensively loaded, adversely affecting performance and availability. The replication technology which means providing multiple resources, e.g. software or hardware components (Maamar et al. 2008), is considered one of the best solutions that would help WS providers to improve the performance and availability of their published WSs (Maamar et al. 2008). The availability ensures that the web service is ready for immediate customer consumption (W3C Working Group Note 2003). For example if a single service becomes overloaded or fails, other copies will be initiated in order to answer the customer requests. Performance is concerned mainly with decreasing the response time of the incoming requests, through balancing the requests among the available replicas.

The replication process clearly requires extra space and extra processing time. Thus, it should be established in a way that it can reduce the number of deployed replicas and continuously remove unused ones. Two important factors should be monitored when checking the status of running replicas: 'service load’ and 'service prediction load’. Of course, when the service is overloaded it requires other copies to balance the load and hence, it might consume unexpected large number of resources. So it is necessary to monitor and predict the incoming load as well as the involved resource utilization.

A web service might be available and unloaded, in which case the consumer would try to access it with no response. This possibly occurs in the case of composite services, where the target web service may be dependent on other unavailable or overloaded web services. Therefore, the replication process should consider all web services either those invoked alone or those involved in a composition process. Also, it should consider the interactions and dependencies among the original web services and their replicas in addition to the other involved web services in the composition process.

In this paper, we discuss and develop an adaptive replication framework for automatically monitoring and replicating basic or composite web services when they fail or become overloaded. The suggested framework conducts composition by orchestration, where a component, called the orchestrator, controls the composition activity. The framework adaptively improves the availability of the published services. It also dynamically enhances the performance of the running web services during *runtime*. Furthermore, it predicts the load of the main running services. Finally, it provides system scalability through facilitating the addition of extra web services or resources as required by the established environment.

The rest of the paper is organized as follows. Section 2 shows the related work. Section 3 introduces a full description of the suggested adaptive replication framework. Section 4 explains the workflow of the proposed framework. Section 5 shows Implementation and Performance. Finally, conclusion and future work are presented in section 6.

## Related work

The replication process has been classified in several ways according to the involved components and their characteristics including requests, replicas and hosts. For instance, which QoS parameters should be considered to select a particular server in order to host a new replica when the original web service is failed or being overloaded. The work in (Salas et al. 2006) partitioned the replication process, with respect to the interaction among replicas and requests, into three types: active, passive and semi-active. In the active type, the consumer broadcasts his/her requests to all available replicas. The one which manipulates the request first would respond to that request and interact with the consumer directly. In the passive type, one of the available replicas would be elected to be the primary replica to communicate with incoming requests and be responsible for updating all other available replicas of the data interchanged and the operations carried out with the consumers. Another primary replica will be determined if the original one fails. Unlike the active and passive types, the semi-active type allows all available replicas to process the incoming requests simultaneously. In the meantime, a master replica is specified to manage and control the communications among the consumers and replicas.

The proposed research in (May et al. 2009) studied the replication process and suggested three other strategies: parallel, serial and composite strategies. The parallel strategy works just like the active type in (Salas et al. 2006). However, in the serial strategy, the consumer is notified, by an ordered list, of all available replicas which can manipulate the incoming request as needed. Therefore, the consumer communicates with the first replica in the defined list; if the selected replica fails or does not answer the request during a certain period of time, the consumer selects the next replica in the list and starts binding with it. Finally, the composite strategy is a combination of the two mentioned strategies, aiming to improve the interaction between the consumers and the available replicas, taking the network traffic status into consideration.

The suggested study in (Zheng and Lyu 2008) expands the replication types in (Salas et al. 2006) into nine strategies, by considering other constraints including time redundancy that might have an impact on the replication process. They can be considered as a combination of the active and passive replication types. For example, the time redundancy constraint enables the consumer to tolerate faults by sending several requests to a specific replica when it encounters failure or overloading till he/she obtains an answer in a reasonable period of time.

In (Liu et al. 2011), a Genetic Algorithm (GA) is proposed to produce a near-optimal replication scheme via a Directed Acyclic Graph (DAG), which is used to discover all possible replication schemas.

In this work, an adaptive replication framework is suggested to allow a consumer to pass requests to a specific replica in the same way as in the passive replication case. However, when the target replica fails or becomes overloaded, the framework replicates the original service into another server during runtime and then forwards the consumer’s requests to the new replica automatically. Notably, the suggested framework considers only replicating the target services without replicating the incoming requests.

One of the main aims of replication is to provide availability as discussed in (Wenbing 2007; Liang and Bin 2010; Ooi et al. 2012). In addition, it also improves the performance of the entire system that the different Web services operate on (Keidl et al. 2003; You et al. 2009; Björkqvist et al. 2012).

In paper (Wenbing 2007), a fault tolerant framework is designed and implemented for managing replication. The suggested framework dynamically supports switching between replication and non-replication operation modes depending on the Web service status and in particular its availability and reliability.

A framework has been proposed in (Liang and Bin 2010) for accomplishing a changeable-location replica at runtime. Fundamentally, the suggested framework establishes an adaptive re-selection of a higher priority service when the main service or host fails. The service priority order is organized based on considering some QoS constraints including the network availability as well as the host machine performance, in addition to the execution time and reliability of the services. The main disadvantage of this framework is that it does not consider any other crucial metrics within the replica re-selection process such as the service or host load.

In (Ooi et al. 2012), a dynamic replication framework is proposed for enhancing the services availability and performance using a hybrid set of artificial intelligence techniques including Neural Networks (NN) and Fuzzy logics. A simulation environment to study the suggested framework with respect to different circumstances such as placing and removing the involved resources dynamically, is presented there. However, the work does not address the replication process in a composite service environment.

The framework in (Keidl et al. 2003) is designed to enhance the performance of the published Web services. Basically, it studies a dynamic Web service selection and replication processes at runtime, particularly when the host becomes unavailable or overloaded. However, it does not address the hosts’ selection strategies. Moreover, it only replicates the regular or basic services, not the composite services.

The framework suggested in (You et al. 2009) aims to improve the QoS parameters of the composite services by deploying multiple replicas on the idle servers to overcome the main server problems such as failure or overloading. Two main components have been defined: Longest Delay Service Component Selection (LDCS) for services failure monitoring and Maximum Available Capacity Path (MACP) for investigating the involved servers. When LDCS detects that a particular service is bottlenecked or overloaded, the framework selects another server for hosting a new replica from some specific available servers utilizing MACP.

The authors in (Björkqvist et al. 2012) introduce a policy for detecting the number of replicas required for accomplishing a specific composition process. The policy reduces the operational cost and minimizes the response time of the running composition applications. The number of required replicas is determined on the basis of the predicted workload. The main drawback of the work proposed in (You et al. 2009; Björkqvist et al. 2012) is that both do not discuss the case when the failure occurs on the host side.

The work in (Mohamed-K and Mohamed-H 2012) is similar to the work in (Mohamed et al. 2012); however, instead of using linear aggression for predicting the load, it uses they deployed a generalized time series technique, without providing details about that algorithm.

Several researchers restrict the replication management process to just selecting the optimal web server for hosting the needed replica, as is the case in (Mehmet et al. 1998; Silva and Mendonca 2004; Björkqvist et al. 2012). The work in (Mehmet et al. 1998) explains six server selection algorithms like Fixed, Ping, Hops, Parallel, Probabilistic and Refresh. The research in (Silva and Mendonca 2004) demonstrates five server selection policies as Random Selection, Parallel Invocation, HTTPing (or Probe), Best Last, and Best Median. Finally, the authors in (Björkqvist et al. 2012) used two replicas selection algorithms, namely Distributed Shortest Queue Selection (D-SQ) and Distributed Round Robin Selection (D-RR).

The authors in (Marco 2001) classify the sever selection algorithms into three applied policies: static, statistical, and dynamic. In the static policy, the server selection is based on such resource parameters as the number of hops, connection bandwidth, and server architecture. The statistical policy considers the competition among the servers depending on pervious collected performance metrics such as latencies and bandwidths. Finally, the dynamic or runtime policy elects the optimal server based on the current network status in addition to the server conditions such as server workload and response times. In the present paper, we use a similar technique to the last policy dynamic selection algorithm (Marco 2001) in which the server selection process is dynamically established during runtime by considering some QoS parameters such as availability and performance.

## The adaptive replication framework

This section explains the main components of the adaptive replication framework which is structured for handling and managing the basic and composite Web services running in a SOA environment. In an earlier work (Mohamed et al. 2012), we proposed and briefly described an adaptive replication framework for dynamically replicating a single web service, the basic type, when a service approaches failure or overloading. In the present work, the proposed framework is designed for monitoring and replicating the running basic or composite web services when it fails or becomes overloaded. In particular, the suggested framework conducts only the orchestration composite type in which a component, the orchestrator, controls the composition process among a set of s basic web services integrated together for implementing a certain business process. In other words, the orchestration composite type would be considered as a generalization of the basic type as there is no mutual dependency among the invoked web services in a composite business process.

The proposed adaptive framework improves the availability of the published web services through reacting to failures on the servers of the consumed services. The adaptation is realized by automatically replicating the failed services on another server. Then, the incoming requests would be transparently redirected to that server. Remarkably, the requests of a consumer will not be replicated; they will just be forwarded to the free replicas. The framework enhances the performance of the published services by using a centralized dynamic load balancing process (Alakeel 2010). The dynamic load balancing process is needed to make request balancing decisions at runtime. And the load balancing process, being centralized, will execute the utilized load balancing algorithm, e.g. the Round-Robin algorithm (Silberschatz et al. 2010), as a single service, called Load balancer. For example, if the server that publishes a particular web service becomes overloaded at some point in time, the suggested replication framework will make the required decision immediately to replicate the published web service on another server and balance the incoming requests between the original and the copied services.

### The proposed adaptive replication framework

**Figure 1:**
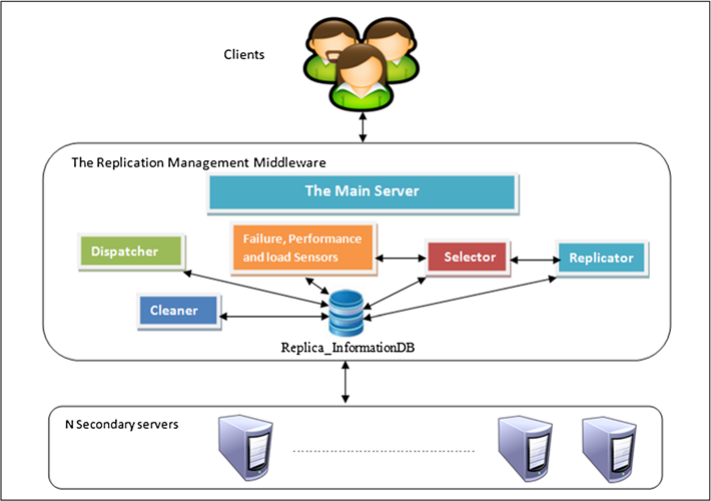
**The adaptive replication framework.**

Figure 1 shows the suggested adaptive replication framework. It includes three layers: the clients or Web service consumers, the replication middleware and the server layer (Mohamed et al. 2012).

The Server layer:

1. *The Main Server (MS)* is located at the replication middleware layer, and is responsible for managing the incoming client requests to the consumed services, and then passing them to the suitable secondary servers that host the required services.
2. *The Secondary Servers (SSs)* are located in the Server layer, and are responsible for processing the client requests and then passing the results to the main server. The number of secondary servers that process client requests is not fixed but changes over the time depending on the current loads. The main server forwards requests to a single replica when the original server load is less than a predefined threshold set by the service provider; otherwise it balances the requests between two replicas.

#### The main server components

A- A number of s*ensors* are used to observe changes that might occur in the secondary servers. These sensors are:

1. Failure sensor: checks the status (available or failed) of secondary servers. It sends ping requests to each secondary server periodically (typically, every 20 seconds). If the server does not respond, the server status will be changed to fail.
2. Performance sensor: collects information about the secondary servers such as the capacity of memory and hard disk in addition to the processor type. In the secondary server reservation phase, the framework sends automatically two web services to each secondary server: the first collects capability information, such as hard disk size, memory size and processor type, and the second collects the CPU load on the server.
3. Load sensor: gathers the CPU usage of the secondary servers periodically (typically, every 20 seconds). Like the Failure sensor, it stores the information in the Replica_InformationDB database as depicted in Figure 1.

B- A *Selector* analyzes the sensors outputs. Consequently, it sorts the available Secondary servers descendingly, from best to worst, according to the following performance metrics:

- Server Status, which includes the following two components:
  1. Availability: if a failure sensor detects a fault on a secondary server, the server will be deleted from the selection list.
  2. Type (Permanent or temporary): a server is temporary if it is, for example, rented or cannot be used for a specific period of time; otherwise the server is permanent.
- Server Load, which includes the following three components:
  1. The current average CPU usage of the available secondary servers for the last minute. Naturally, low-load servers get a higher rank than heavy load servers.
  2. Future load on the available secondary servers, which can be predicated using linear regression analysis (Montgomery and Runger 2007). Logically, servers with low predicted loads get a higher rank than those with heavy predicted loads.
  3. Scheduling of secondary servers. Obviously, a server that has a free schedule is better than one that does not.
- Server capabilities, which includes the following component:
  1. Capabilities of the secondary servers such as the processor type, memory capacity and hard disk capacity. In our simulation, all secondary servers have the same capabilities, so this factor is ignored in the calculations.

The selection process is mainly accomplished by calculating a value equal to the sum of the pervious listed parameters by utilizing Equation 1.1

Where *R*_*s*_ is a number describing the server status (e.g. Available_permanent= 2, Available_rented = 1 and Failed = -1), *R*_*l*_ is a scaling number representing the server load and *R*_*c*_ is a normalized number identifying the server capabilities. The server with the highest value is selected first, then the second, and so on.

**Figure 2:**
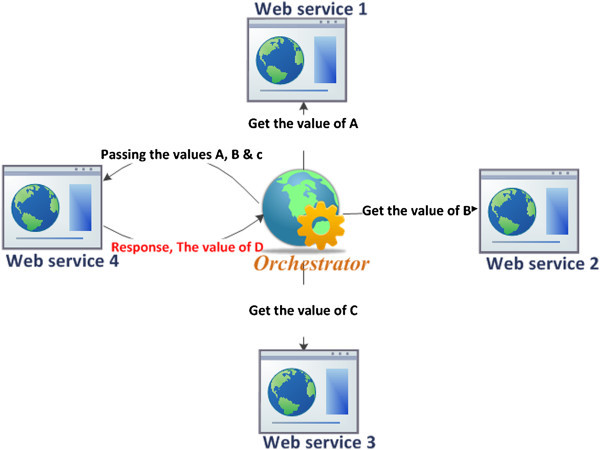
**Example of web services composition with orchestration.**

C- *Dispatcher*: It acts as a mediator between the consumers and the secondary servers; it accomplishes the following roles:

1. Responder: exchanges and organizes message traffic between the consumers and Secondary servers.
2. Load balancer: applies the dynamic load balancing process (Alakeel 2010), which is basically implemented through executing three stages: Information, Transfer, and Location (Alakeel 2010). The Information stage is for collecting data including the running and administration data relating to the deployed SOA environment and that accomplished via several sensors such as load, performance, and failure. The main function of the Transfer stage is to transfer decisions issued when some changes have been detected such as a server failure. Finally, the Location stage is assigned for the alternative servers selection process which would be performed based on a hybrid set of QoS metrics, including the server availability, performance and load. Finally, it balances the load of the incoming requests.
3. Orchestrator: the dispatcher acts as an orchestrator or coordinator in the case of the composite web services. It controls the communication and interaction among the WSs constituting a particular business process. For example an orchestrator invokes WS1, WS2 and WS3, then passes the outcomes 'A, B and C’ to WS4 in order to allow WS4 to accomplish its task as seen in Figure 2.

**Figure 3:**
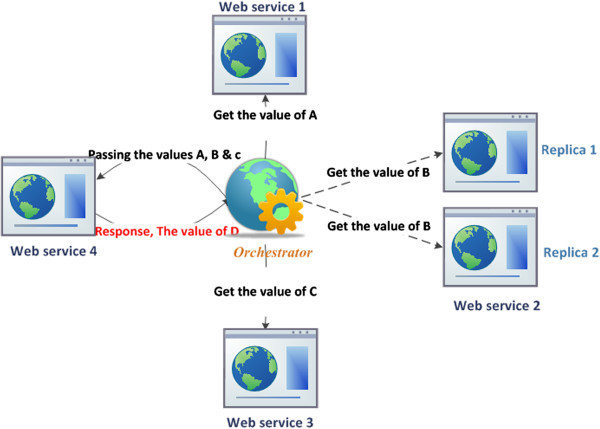
**Example of web services composition with orchestration.**

Note that when WS2 which is involved in a specific composite process is overloaded, the dispatcher or the Orchestrator, in this case, acts as the Load balancer to control and distribute the incoming messages among the replicas shown in Figure 3. More details about the replicas management process within the WSs composite case are given in the following section.

D- *Replicator*: It checks the server availability and performance, assumed in this work to be every 20 seconds. Once it detects that a server stopped working, it replicates the web services files on a secondary server automatically. Hence, the load sensor obtains the server load periodically, and then the average load of secondary servers is updated. The replicator checks this update and also ensures the server availability to take the appropriate action. Generally, two approaches have been used to control access to replicated resources on the Internet: the server side and client side approaches (Silva and Mendonca 2004). The Server side approach is utilized in cases where server holding replicas are connecting and communicating together physically or under the same administration, as is the case in server clusters.

The second approach basically considers the client’s characteristics and preferences to access the needed replicas. Thus, the client side approach is mostly suitable in cases where replicas are geographically dispersed. In the adaptive replication framework proposed in the present work, the server side approach is utilized for controlling and managing the replication process where the clients access the replicas through sending requests and receiving responses.

E- *Cleaner:* It examines the usage status of all secondary servers and their embedded WSs. In other words, it checks the secondary servers that have inactive replicas which have not received any messages during a certain period, assumed to be every 60 seconds. Therefore, it deletes the inactive web services from the secondary servers and changes the status of the servers to be “unused” if it does not have any active replicas.

## Workflow of the adaptive replication framework

The workflow of the suggested replication framework can be divided into two phases: Reservation and Runtime. The Reservation phase is for collecting information about the involved Secondary servers and the running web services. The Runtime phase is for invoking the replication process during *runtime* according to particular QoS metrics including performance and availability.

### Reservation

In this phase, the Main server records and maintains the basic information about Secondary servers and web services such as server name and IP in addition to the web service name and URL.

At the beginning, the servers are registered manually and then monitored dynamically by the failure sensor. Periodically, Equation 1 is recalculated by the selector to update the servers’ selection list.

#### Secondary servers reservation

The server reservation phase includes saving the basic information about the available Secondary servers in Replica_InformationDB. The information look likes

- Name, IP addresses, FTP information (i.e. FTP Name, username and password). That information is needed in order to transfer the copied WSs to the selected secondary servers.
- Status (permanent or temporary), and if it is a temporary server, the date and timestamp of the renting period will be registered.
- Schedule list: it registers the date of the possible activities that may make a server loaded.

#### Web service reservation

The Web services reservation phase includes saving the basic information about the web service on the Replica_InformationDB. The information look likes:

- Web service Name
- Initial position of the web service 'secondary server IP’.
- Web service file: it used by replicator when the master replica fails or becomes overloaded.

### Runtime

There are three scenarios for implementing the replication process:

- Static scenario: there is no replication, only one service executes the consumer request.
- Adaptive scenario: in this scenario, the replication is done automatically when the service fails or overloaded.
- Adaptive with prediction scenario: is the same as the previous scenario except that it is including the prediction load capability.

Of course, some other scenarios might be located between the main discussed in this work including the regular load balancing scenario. In such a scenario, the servers and the embedded replicas are fixed and the incoming requests are distributed and manipulated among them whether the detected load is high or low. In this work, we are only interested in the dynamic environment in which the load, servers and replicas change periodically.

A full comparison between these scenarios is demonstrated in the Implementation and Performance section.

#### The static scenario

**Figure 4:**
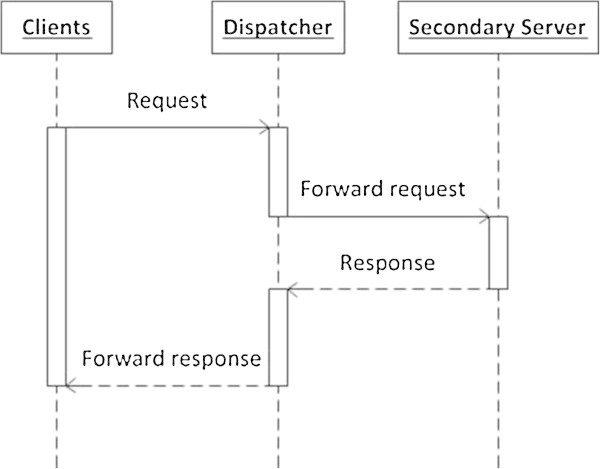
**The sequence diagram of the static scenario.**

In this scenario, the dispatcher determines some server(s) to process the client requests. If the server(s) and the hosted web services are working properly, no action would be taken. Figure 4 explains how this scenario works.

- A client sends a request to the Dispatcher.
- The dispatcher forwards the request to the secondary server(s) for processing.
- The secondary server(s) processes the client requests and sends the response to the Dispatcher.
- The dispatcher sends the response back to the client.

#### The adaptive scenario

The functionalities of the Dispatcher, Replicator and cleaner within the adaptive scenario are demonstrated below.

**Figure 5:**
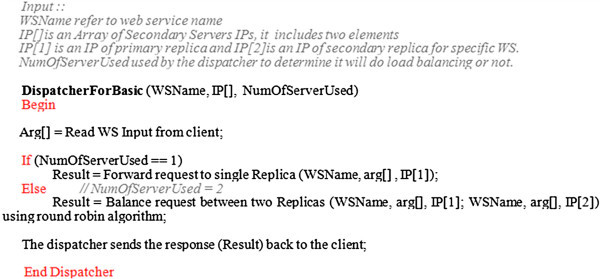
**The dispatcher algorithm for a basic web service.**

**Figure 6:**
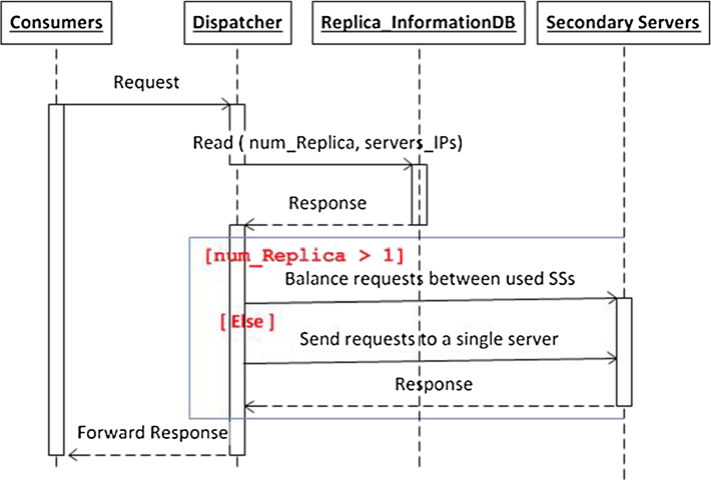
**The sequence diagram of the dispatcher scenario.**

- A client sends a request to the dispatcher.
- The dispatcher reads the number of replicas and server IPs used from the Replica_InformationDB database. If the number of replicas is 1, the dispatcher forwards the request to a single secondary server. If there multiple copies of the same service, the dispatcher balances the client request and other incoming requests between these replicas using the round robin algorithm.
- The Secondary server(s) processes the incoming request (s) and sends the response to the dispatcher. Then the dispatcher sends the response back to the client. The algorithm in Figure 5 shows how the dispatcher works. Also, the sequence diagram in Figure 6 summarizes the main dispatcher functionalities.

As seen in Figure 6, the dispatcher uses the information stored in Replica_InformationDB to decide when it should replicate a loaded service and then balance the load between the original service and its replica(s). The replicator is the responsible component for specifying the replication time, as explained later. In this framework, the three components: dispatcher, replicator and cleaner work in parallel.

**Figure 7:**
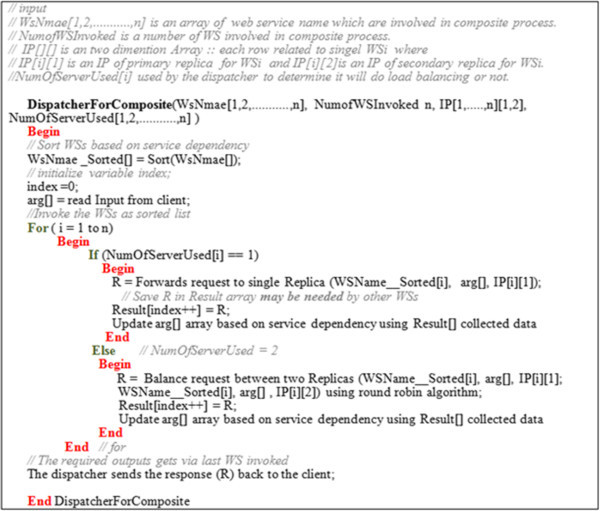
**The dispatcher algorithm for composite web services.**

In the case of composite web services, the dispatcher acts as an orchestrator to coordinate the different web services that are involved in the WSs composition process. First, the orchestrator reads the information available about all the WSs registered in Replica_InformationDB. Second, it calls and collects the outputs from WSs as shown in Figure 2. Finally, it forwards these outputs to a specific WS in order to accomplish its task. The algorithm in Figure 7 formalizes the preceding steps.

The replicator checks the used secondary server(s) every 20 sec. If the secondary server fails, the replicator calls the selector to choose the best available secondary server (s). Then it replicates the original web service on the selected server(s). Also, it updates Replica_InformationDB by the writing the new IP address of replica(s).

**Figure 8:**
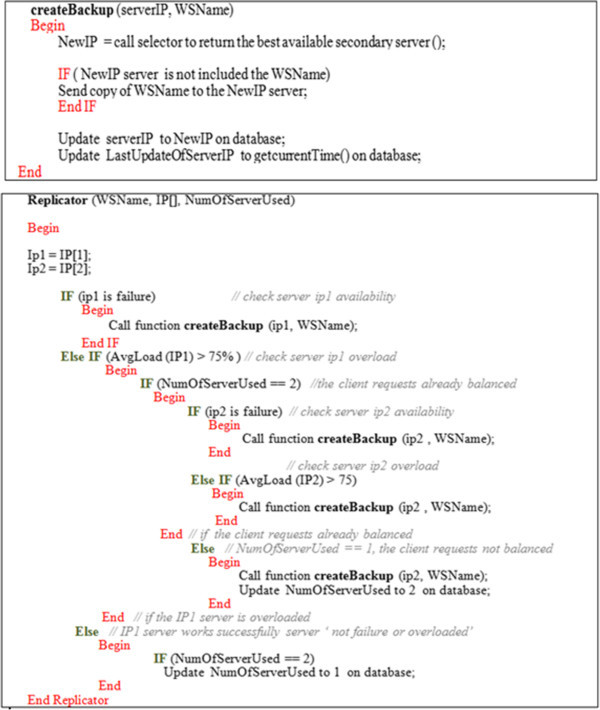
**The replicator algorithm for a basic web service.**

**Figure 9:**
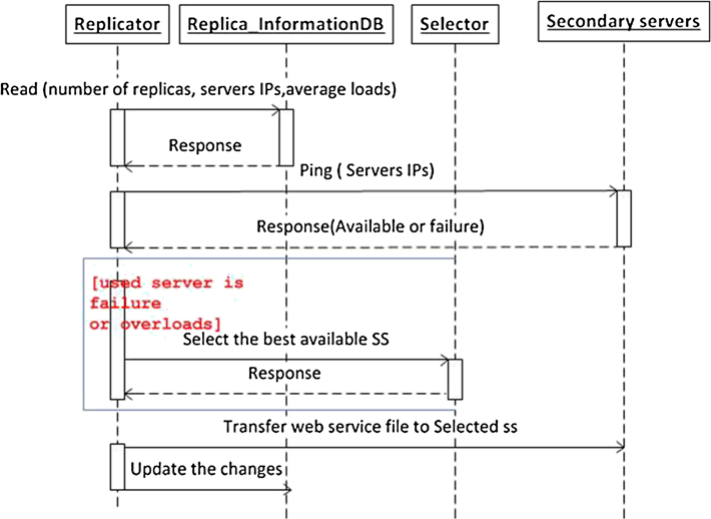
**The sequence diagram of the replicator scenario.**

The algorithm in Figure 8 formalizes how the replicator works. Also, the sequence diagram in Figure 9 summarizes the main replicator functionalities.

In the composition case, the replicator checks the availability and performance of the WSs involved in implementing the composition process. It should be noted that in the orchestration composite case, none of the involved WSs calls any of the others. However, the Orchestrator is the component which manages the communications among those WSs and consequently their replicas as well. In other words, the invoked web services are fully operated and controlled through a relationship loosely coupled with the central Orchestrator.

**Figure 10:**
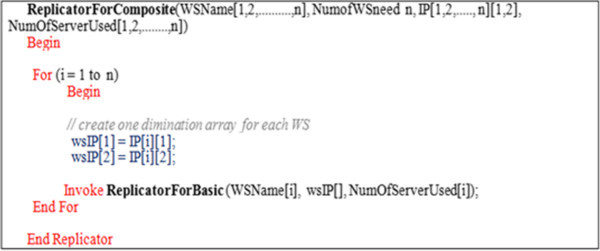
**The replicator algorithm for composite web services.**

The orchestration composite replication in this case would be considered repetition n times of the Basic replication process with respect to the *n* loaded or failed WSs embedded in establishing the orchestration composite case as shown in Figure 10.

After checking the status (i.e. active or inactive) of the existing replicas, the cleaner removes all unneeded replicas. The inactive replica is the WS that has no longer in call by the consumer or any other components. To investigate that, the following statement is applied:

**IF** currentTime > LastUpdateOfServerIP + ProcessingTime

**THEN** Replica_Status = Inactive

**Else** Replica_Status = Active

Where LastUpdateOfServerIP is the last time in which the Replicator changes the status of the host server for that replica from 'used’ to 'unused’. The ProcessingTime is the period needed by the host server to process consumers’ requests.

**Figure 11:**
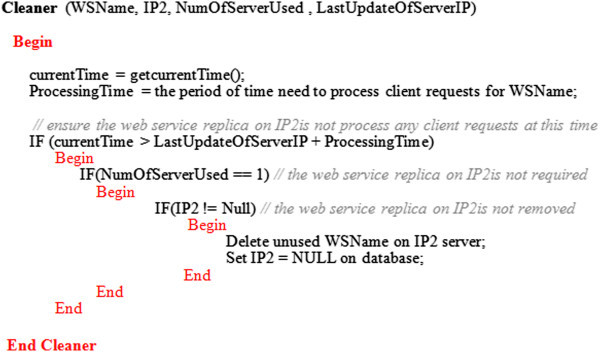
**The cleaner algorithm for basic web services.**

**Figure 12:**
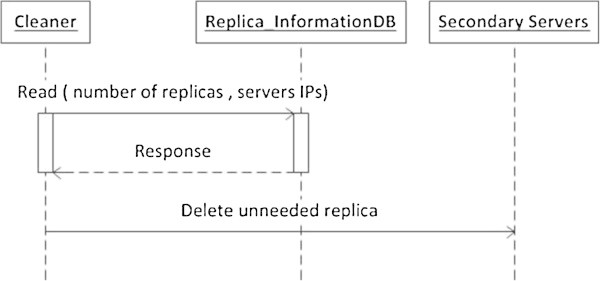
**The sequence diagram of the Cleaner scenario.**

The algorithm in Figure 11 formalizes how the cleaner works in basic web services case. Also, the sequence diagram in Figure 12 summarizes the main cleaner functionalities.

**Figure 13:**
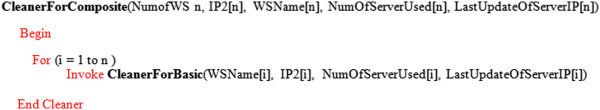
**The cleaner algorithm for composite web services.**

In the composition case, the cleaner deletes all inactive replicas 'WSs’ involved in the composition process. The algorithm in Figure 13 shows the steps that the cleaner follows to delete the inactive replicas within the composition case.

#### Adaptive with load prediction scenario

In this scenario, the dispatcher, selector and cleaner mechanisms for basic and composite WSs work as explained in the adaptive scenario. However, a prediction utility is added to the replicator in order to predict the incoming load in a specific day using the following factors:234

- Linear regression can be used to explain the relation between x (the load of the current day) and y (the load of the next day) by using Equations 2, 3 and 4. Notably, it might be known that the server load follows a nonlinear statistical model (Montgomery and Runger 2007).

**Figure 14:**
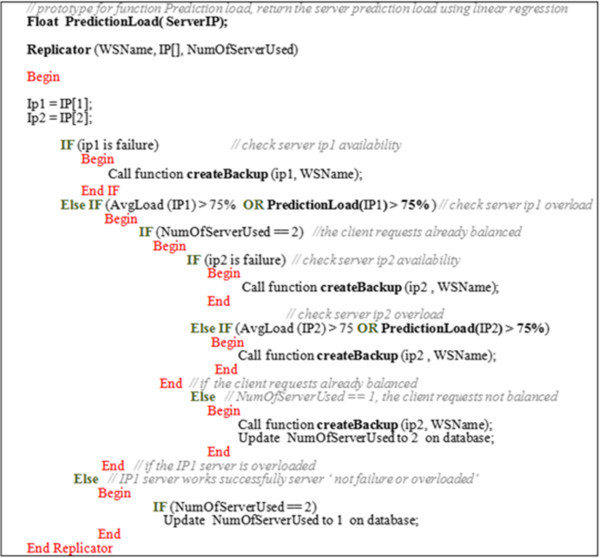
**The replicator algorithm for basic web services.**

Where  and  denote average. These equations will be verified by an example in the following section. The algorithm in Figure 14 demonstrates the way in which the replicator works.

## Implementation and performance

In (Mohamed et al. 2012), we introduced a case study for demonstrating the Basic replication workflow. Hence, it would be worthy to only focus here on the scenario of the Composite WSs replication. In this section, we will discuss a case study for showing the Composite replication workflow in order to evaluate the proposed framework.

The composite replication case study is described as follows. In our University, at the beginning of each academic year, the students often access the University home page, hence the interconnected University servers, to know whether they have been accepted in the dormitories. A student is accepted if and only if he/she satisfies the following conditions:

- The grade in the previous year must be C or higher.
- The tuition fees have been fully paid.
- The medical examination shows no infectious diseases.

In this scenario, at least three different web services are running to handle the above conditions; they are deployed on different web servers which are established as follows:

- The academic server located in each faculty where the student registered. This server manages regulations A and B through a published web service named 'academic_WS’.
- The medical server located inside the University hospital in which the student establishes his annual medical examination. This server conducts regulation C by a particular web service called 'medical_WS’.
- The Dormitories server located in the main University Dormitory in which the previous regulations (A, B and C) are passing to in order to check the acceptance status of each registered student through consuming the Dormitories web service defined as 'Dormitories_ws’.

**Figure 15:**
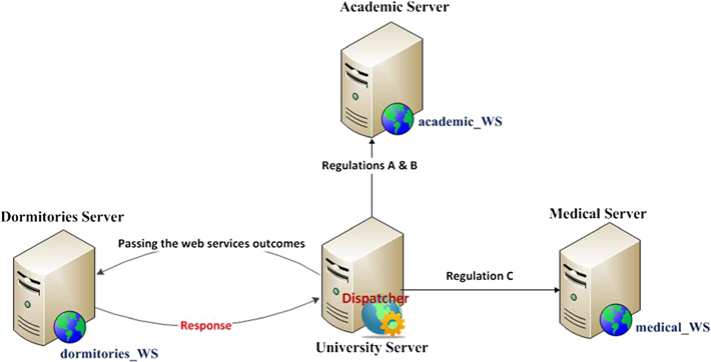
**The orchestrated composite dormitories web services.**

The University server is responsible for controlling and monitoring the interaction among the previously mentioned servers as depicted in Figure 15.

Each student is usually asked to submit his/her national ID or University ID, and then a specific data verification process will be accomplished through the suggested replication framework. First, the dispatcher invokes the 'academic_WS’ to check the status of regulations A and B; the tuition fees and academic report of each registered student. Then, the dispatcher calls the 'Medical_WS’ to verify regulation C, the student health report. Then, the dispatcher passes all outcomes of the running web services to the 'Dormitories_WS’ in order to determine whether the student would be accepted or rejected.

*In the Static scenario 'without Replication’:* If any of the published web services, such as 'academic_WS’, or of the host servers, such as the academic servers fails, the student will not get a response. Furthermore, if any of the host servers is overloaded, the student has to wait longer to get a response because, in that case, no load balancing technique is supported.

*The Adaptive Replication scenario*: The University server manages the web services replication process. If it detects the failure of a web service or a host server, the framework automatically replicates the consumed service on another server (i.e. a Secondary server) located inside the University campus based upon certain Service-Level Agreements (SLAs) including their performance and availability. Moreover, if the University server detects the overloading of a web service or host server, the framework automatically replicates the consumed service on another server then uses the Round Robin load balancing algorithm (Silberschatz et al. 2010) to balance the requests on the replicas.

**Figure 16:**
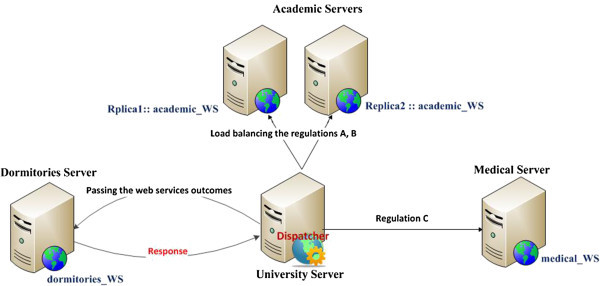
**The orchestrated composite dormitories web services with a replica.**

*Adaptive with a load prediction scenario:* Adding to the previous case, a prediction utility using linear regression is employed in order to save the replica deployment time. In this experiment, we assume that the academic server is overloaded by the incoming requests, so the replicator transfers the academic_WS to another academic server located in the University campus (i.e. located in another faculty inside the campus) and balances the incoming requests between the two servers as shown in Figure 16.

The following example explains how the server load prediction works. The Load sensor collects the historical data about the load of all available servers in the campus, for two weeks. This data is saved in a particular database virtualized as a queue that is updated daily, with the newest value added at the tail of the queue and the oldest value removed from the head (As a shifting process). This shown in Table 1, where the odd days (i.e. 1st, 3rd May and… etc.) are expressed as *x* with respect to the mentioned equations in Section 4 and the even days (i.e. 2nd, 4th May and… etc.) are presented as *y* with respect to the same equations in Section 4.

**Table 1:** **The history average load of the target academic server**

Day	Saturday 1 - 5	Sunday 2 - 5	Monday 3 - 5	Tuesday 4 - 5	Wednesday 5 - 5	Thursday6 - 5	Friday 7 - 5
The academic server average load	62	80	50	45	72	52	39
**Day**	**Saturday 8 - 5**	**Sunday 9 - 5**	**Monday 10 - 5**	**Tuesday 11 - 5**	**Wednesday 12 - 5**	**Thursday 13 - 5**	**Friday 14 - 5**
The academic sever average load	67	69	55	50	85	45	30

If the load of the target academic server for the day comes directly after the two monitored weeks (i.e. Saturday, May 15th) it is measured and expressed as *x*= 60 requests then the predicted load of the academic server for the day after (i.e. May 16th) which is presented as *y* as will be calculated.

Remarkably, we assume the load will be predicted daily because it is more suitable for our case study. For example, at the beginning of the academic year, the Dormitories server will understandably become overloaded during the entire day.

Table 2 involves the needed values to calculate *β*_1_, *β*_0_ and *y* as explained in section 4.3 using the Equations 2-4

**Table 2:** **The load prediction calculation of the target academic server**

	x	y	xy	x^2^	y^2^				
1	62	80	4960	3844	6400	6.714286	20.86	140.0408163	45.08163265
2	50	45	2250	2500	2025	-5.28571	-14.14	74.75510204	27.93877551
3	72	52	3744	5184	2704	16.71429	-7.143	-119.3877551	279.3673469
4	39	67	2613	1521	4489	-16.2857	7.857	-127.9591837	265.2244898
5	69	55	3795	4761	3025	13.71429	-4.143	-56.81632653	188.0816327
6	50	85	4250	2500	7225	-5.28571	25.86	-136.6734694	27.93877551
7	45	30	1350	2025	900	-10.2857	-29.14	299.755102	105.7959184
Total	387	414	22962	22335	26768			73.71428571	939.4285714

The following experiments were conducted to evaluate the performance of the framework using the previous scenarios (static, adaptive and adaptive with load prediction). The test environment in VMware simulation consists of 6 servers: 1 MS, 5 SSs and 1 DNS. The DNS server is used also as a secondary server. The capabilities of these servers are established in Table 3. All suggested secondary servers are academic servers running inside the University.

**Table 3:** **The capabilities of simulation servers**

Capabilities	MS	SSs	DNS
**HD**	40 GB	40 GB	40 GB
**CPU**	Intel® core ™ Num of core using by VMware 1		
**Memory**	500 MB	384 MB	384 MB
**OS**	Microsoft windows server 2003		
**Language**	PHP scripting language		-

The experiments were conducted using *ApacheBench* Version 2.0.40-dev <$Revision: 1.146 $>apache-2.0 by passing parameter such as:

- The number of requests that are passed to the University web page. In this experiment, the requests are passed to the dispatcher component, which is located on the University server.
- The concurrency level is the number of concurrent requests passed to the web page at the same time. For example, if we want to run 1000 requests and the concurrency level is 10, then the requests will be sent over 100 times. Of course, there is positive correlation between concurrency level and server load, in the sense that when the concurrency level increases, the server load also increases.
- The web page URL required for testing 'Dispatcher web page’.
- Number of periods required to be tested and the time in seconds between these periods.

The experiment is accomplished by passing a number of requests equal to (150, 300, 450 and 600) representing the number of target students who would like to submit in Dormitories. Also passed are their concurrency levels (1, 3, 6, 9 and 12) indicating the number of students who submit their requests simultaneously. Then, the response time and throughput are calculated at each concurrency level. At level 1, say, the response time is calculated by summing the response time values resulting from passing (150, 300, 450 and 600(. At the other levels the response time will be calculated similarly.

**Figure 17:**
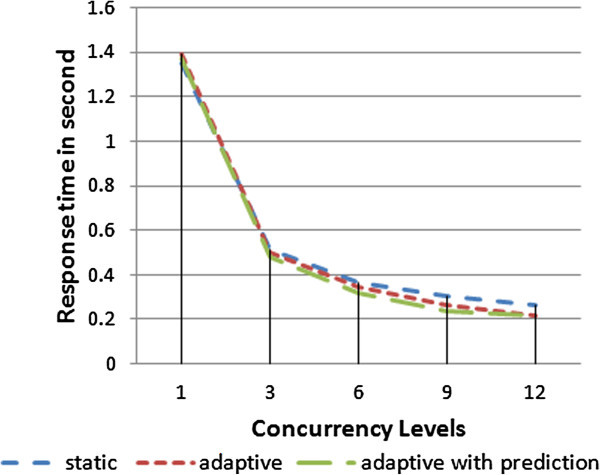
**The response time of running 1500 requests.**

Figure 17 demonstrates the charts for the above experiment with the three different scenarios: static, adaptive and adaptive with prediction. As shown in Figure 17, the concurrency levels (1and 3) reflect the low load of requests, the concurrency level (6) reflects the average load of requests, and also (9 and 12) reflect the high load of requests; depending on the used server capabilities.

**Figure 18:**
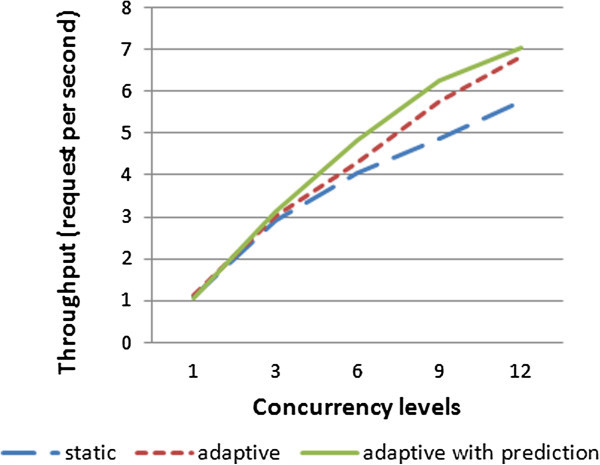
**The throughput of running 1500 requests.**

The performance order of the previous approaches shown in Figures 17 and 18 from best to worst is adaptive with load prediction property, adaptive replication and static approaches. Note that when the concurrency level is increased above 3 requests per second “low load case”, the gap between static and adaptive is increased, whereas the gap between adaptive and adaptive with prediction is decreased. When the concurrency level equals 1, i.e. a request passing at a time, the response time and throughput of the three techniques approach the same value. In this case, there is no need to balance the incoming requests and typically the adaptive scenario would act as the static one by forwarding requests to a single replica.

If the concurrency level is 3 or 6, the adaptive replication technique pass more requests to a single replica, so that the average load takes longer time to reach to the threshed (assumed 75%). Thus, it becomes nearer to static and far from adaptive with load prediction property.

When the concurrency level is 9 or 12, the adaptive replication technique balances the requests on the two replicas because the average load reaches the threshed quickly (75%). So it becomes nearer to adaptive with load prediction property and far from static approach.

## Conclusions and future work

In this paper, an adaptive replication framework for basic and composite web services is introduced and described. The framework aims to improve the web services availability. It also minimizes the response time through supporting an adaptive replication of the consumed web services, considering the environment changes that might occur at the service provider side such as failure or overloading. In particular, the suggested framework studies the orchestration composite type through managing the interconnection among some regular web services and replicates the poorly-operated service.

Moreover, the framework includes a particular utility for predicting the future load on the servers hosting the original copies of the web services. The prediction helps decrease the time of outsourcing the replicas on other available servers.

A case study of accomplishing the replication process for an orchestrated composite web service is presented. The associated experiments study the proposed framework in three different scenarios: static 'without replication’, adaptive and adaptive with load prediction property, and measure the response time and throughput. The shown outcomes prove that the framework operates most efficiently when it runs with the adaptive with prediction property mode.

For *the future*, we plan to apply the adaptive replication framework on choreographically composite web services, where no central component controls the interaction between Web services, but rather the Web exchanges messages directly among them.

Furthermore, we aim to apply a partially adaptive replication over the published coarse granular web services, where a service encapsulates different business processes or operations. We believe that it would be better to replicate the service operations which often have a high load in the form of a standalone web service to guarantee the privacy of the main service as well as to enhance the interoperability and granularity of the composite service process

Moreover, we plan to utilize other prediction load algorithms by accomplishing another robust statistical prediction technique for managing properly all types of data sets, including linear and nonlinear sets. Likewise, we are willing to improve the server selection techniques through considering the consumer requirement preferences.

Finally, we plan to apply the adaptive replication on another distributed system including a cloud computing platform.
